# Metabolome and RNA-seq Analysis of Responses to Nitrogen Deprivation and Resupply in Tea Plant (*Camellia sinensis*) Roots

**DOI:** 10.3389/fpls.2022.932720

**Published:** 2022-08-26

**Authors:** Wenluan Xu, Jing Li, Luyu Zhang, Xuyang Zhang, Hua Zhao, Fei Guo, Yu Wang, Pu Wang, Yuqiong Chen, Dejiang Ni, Mingle Wang

**Affiliations:** ^1^Key Laboratory of Horticultural Plant Biology (Ministry of Education), College of Horticulture and Forestry Sciences, Huazhong Agricultural University, Wuhan, China; ^2^Key Laboratory of Urban Agriculture in Central China (Ministry of Agriculture), College of Horticulture and Forestry Sciences, Huazhong Agricultural University, Wuhan, China

**Keywords:** *Camellia sinensis*, nitrogen nutrition, RNA-seq, metabolomics, secondary metabolites

## Abstract

Nitrogen (N) is an important contributor in regulating plant growth and development as well as secondary metabolites synthesis, so as to promote the formation of tea quality and flavor. Theanine, polyphenols, and caffeine are important secondary metabolites in tea plant. In this study, the responses of *Camellia sinensis* roots to N deprivation and resupply were investigated by metabolome and RNA-seq analysis. N deficiency induced content increase for most amino acids (AAs) and reduction for the remaining AAs, polyphenols, and caffeine. After N recovery, the decreased AAs and polyphenols showed a varying degree of recovery in content, but caffeine did not. Meanwhile, theanine increased in content, but its related synthetic genes were down-regulated, probably due to coordination of the whole N starvation regulatory network. Flavonoids-related pathways were relatively active following N stress according to KEGG enrichment analysis. Gene co-expression analysis revealed *TCS2*, *AMT1;1*, *TAT2*, *TS*, and *GOGAT* as key genes, and TFs like MYB, bHLH, and NAC were also actively involved in N stress responses in *C. sinensis* roots. These findings facilitate the understanding of the molecular mechanism of N regulation in tea roots and provide genetic reference for improving N use efficiency in tea plant.

## Introduction

Tea plant [*Camellia sinensis* (L.) O. Kuntze] is widely cultivated in more than 60 countries worldwide and tea plantation area has exceeded 5.3 million hectares in 2020.^1^ High-yield cultivation of tea plant is based on the basic theories of photosynthesis characteristics, ecological response, transportation, and distribution of carbon (C) assimilates, with a focus on nutrition, fertilization, and soil management. The increase of tea yield is largely attributed to the contribution of fertilizers, especially Nitrogen (N) (Wang et al., 2018). N is not only an important part of life macromolecules such as nucleic acids, but also plays an indispensable role in regulating plant growth and development due to its involvement in the composition of plant hormones, signal molecules, and other essential plant macromolecules. Additionally, effective N can promote the accumulation of secondary metabolites.

In plants, the response to N starvation is a complex regulatory network, primarily including the regulation of root N absorption system and root system architecture (RSA; Bellegarde et al., 2017). The most important regulators in the root N absorption system include signal transduction, transcription factors (TFs), plant hormones, N-related enzymes, and transporters, etc. (Kong et al., 2021). Liu et al. (2017) reported a novel Ca^2+^ signaling triggered by nitrate called nitrate-Ca^2+^-sensor protein kinases (CPK)-Nin-Like Protein (NLP), which is considered to be the center of plant nutrient-growth regulatory network in *Arabidopsis* leaves and roots. Apart from the secondary messenger Ca^2+^, TFs also participate in signal transduction, Chu et al. (2021) discovered a module that interact under nitrate starvation and mediate the signaling of N demand in the system. Moreover, TFs also affect N metabolism, such as N uptake, specifically, abscisic acid (ABA)-related TFs named ABF2 and ABF3 were found to be important hubs among nitrate, ABA signal pathway, TFs, and root development (Contreras-López et al., 2022). Ammonium (${\mathrm{NH}}_{4}^{+}$) and nitrate (${\mathrm{NO}}_{3}^{-}$) can serve as both N sources and signal molecules, being absorbed and transported mainly by the ammonium transporter (AMT) and nitrate transporter (NRT) in plants. AMT and NRT can also adjust RSA by regulating auxin, which is of great significance to improve N uptake efficiency (NUpE; Liu and von Wirén, 2017; Lay-Pruitt and Takahashi, 2020). According to Ortigosa et al. (2021), ammonium may regulate the root development of pine through IAA-cytokinin-ethylene (IAA-CK-ET) phytohormone crosstalk and differential expression of TFs in the apex. Various enzymes and miRNAs were also reported to be involved in N absorption and assimilation, nitrate reductase (NR) activity was shown to be positively related to the level of root auxin, which can promote N assimilation and adjust RSA simultaneously (Fu et al., 2020), while the decreased expression of miR164 and miR167 were closely related to the low N tolerance of rice (Nischal et al., 2012). Collectively, TFs, N-related enzymes and transporters participate in the construction of N starvation regulatory network, which can improve RSA and N use efficiency (NUE), and help plants better adapt to N starvation. It has been reported that plant metabolism could be reprogrammed after N deficiency, and when N was resupplied, anabolism could be gradually restored (Wang et al., 2012), which is an active and orderly process (Tsai et al., 2018).

Proper N fertilization (200 ~ 350 kg/hm^2^) helps improve the yield and quality of tea plant (Chen et al., 2021). Nevertheless, NUE is less than 50% (Baligar et al., 2001). N forms (Ruan et al., 2007; Yang et al., 2020) and supply levels (Dong et al., 2019) could produce significant regulatory effects on the growth and metabolism in tea plant. And the responses to N conditions may vary from low-N tolerance or sensitive tea cultivars (Ruan et al., 2021), sampling tissues (Zhang et al., 2021), and sampling time points (Liu et al., 2020). Effective ${\mathrm{NH}}_{4}^{+}$ absorption, assimilation, transport, and reutilization enabled tea plant’s desire for ammonium (Tang et al., 2020), ${\mathrm{NH}}_{4}^{+}$ can be assimilated into glutamate, and then converted into theanine. Single nitrate exerts a negative impact on tea plant, even performs the same effect as N deficiency in some conditions (Ruan et al., 2007; Fan et al., 2015). Furthermore, the favorable effect of ammonium on tea plant can be weakened by nitrate (Ruan et al., 2019). As a primary nutrient, N stress can influence the metabolism of tea plant, especially C and N metabolism, and further lead to changes of the synthesis of secondary metabolites like theanine and polyphenols in tea plant (Su et al., 2020; Liu et al., 2021a).

The aim of the present study was to investigate the responses of *C. sinensis* roots to N deprivation and resupply and identify the key genes and metabolites involved. The findings of this study will provide a reference for further research on the molecular mechanism of N regulation to improve NUE in tea plant.

## Materials and Methods

### Plant Materials and Treatment

One-year-old cutting seedlings of the tea cultivar “Zhongcha 108” were obtained from Yichang Lichuang Biotechnology Co., Ltd. (Yichang, Hubei, China) and hydroponically precultured in the greenhouse of Huazhong agricultural university (Wuhan, China). After immersion in pure water for 7 days, these seedlings were further cultured with 1/2 tea plant nutrient solution (pH = 5.0; Wan et al., 2012), with the nutrient solution changed every 2 weeks until new roots grew. For low-N treatment, the tea seedlings with the same growth trend were transferred to the nutrient solution with 0.1 mmol·L^−1^ total N (the N content was one tenth of the normal N level, and the other elements remained at the daily level). After 4 days of N deprivation treatment, the normal N level (i.e., 1 mmol·L^−1^ total N) was restored for 2 h. All experiments were carried out with a photoperiod of 12 h light (24 ± 1 °C) / 12 h dark (20 ± 1 °C), 70% relative humidity, and 200 μmol m^−2^ s^−1^ light intensity. The samples untreated and exposed to N deprivation for 4 days were abbreviated as CK and -N 4d, and the -N 4d samples exposed to N resupply for 2 h was abbreviated as Hb 2 h. Finally, the white absorbing roots of tea plant were sampled separately from the groups of CK, -N 4d, and Hb 2 h, immediately frozen in liquid N, and stored at −80°C for transcriptome sequencing and metabolomics analyses.

### Ultra-Performance Liquid Chromatography to Quadrupole Time-of-Flight Mass Spectrometry (UPLC-Q-TOF MS)-Based Metabolomics Analysis

The metabolites of tea plant root samples were extracted as previously reported with minor modifications (Yu et al., 2020). Briefly, fresh root samples (150 mg) from each group were ground followed by extracting the powder in 1.5 ml precooled 75% methanol solution (v/v; containing 7.5 μg/ml D4-acetaminophen as internal standard) for 12 h at 4°C in the dark, then centrifugation at 8000 g (4°C for 2 min) to collect the supernatant, with four replicates for each sample. 100 μl of each extract sample was withdrawn and pooled together to prepare the quality control (QC) samples for evaluating the stability and reproducibility of metabolomic analysis, using 75% methanol solution alone as the control. Non-targeted LC–MS analysis was performed using an Infinity 1290 UHPLC System (Agilent Technologies, Santa Clara, CA, USA) coupled with a Q-TOF/MS instrument (Q-TOF 6520; Agilent Technologies, Santa Clara, CA, USA). Metabolite samples were separated with a Zorbax Eclipse Plus C18 column (2.1 × 100 mm, 1.8 μm, Agilent Technologies, Santa Clara, CA, United States). The elution was performed at 0.3 ml/min flow rate with solvent A (water with 0.1% formic acid, v/v) and solvent B (methanol) at an injection volume of 3 μl. The conditions of LC–MS were set as described by Li et al. (2018). Masshunter Profinder (version B.07.00, Agilent Technologies, Santa Clara, CA, United States) was used for detection and alignment of metabolic peaks, and the contents of detected compounds were estimated by the following equation:


(1) $$\begin{array}{c} \mathrm{Relative}\;\;\mathrm{content}\left(\mu g/g\mathrm{DW}\right)=\mathrm{peak area}\left(C\right)/\\ \mathrm{peak area}\left(S\right)*7.5^{*}1.5/W \end{array}$$

Where C indicates compound; S, internal standard; W, sample dry weight.

### RNA Extraction, Library Construction, and Root RNA-seq

Total RNA was extracted using Trizol reagent (Invitrogen, CA, United States) as instructed by the manufacturer. The quantity and purity of total RNA were determined using the RNA 1000 Nano Lab Chip Kit (Agilent Technologies, Santa Clara, CA, United States) on a Bioanalyzer 2100 system (Agilent Technologies, Santa Clara, CA, United States). Poly (A) RNA was purified from total RNA (5 μg) in two rounds using poly-T oligo-attached magnetic beads. Next, the mRNA was fragmented into small pieces at high temperatures using divalent cations, followed by reverse-transcription of the cleaved RNA fragments to create the final cDNA library following the protocol for the mRNA seq sample preparation kit (Illumina, San Diego, USA). The cDNA library was constructed by the Hangzhou Lianchuan Biotechnology Co., Ltd. (Hangzhou, China) and sequenced by Illumina Hiseq4000 with 150 bp paired-end reads. The root RNA sequencing analyses were performed with two biological replicates.

### Transcriptome Analysis

Clean data was obtained by trimming reads containing adaptors, ploy-N (N > 5%) and low-quality reads from raw data using Cutadapt (Martin, 2011). The valid data was mapped to the genome of “Suchazao” (Wei et al., 2018) using Hisat software (Kim et al., 2015). The quality control of sequencing data was performed using FastQC (version 0.11.6). For functional annotation of all unigenes, all assembled non-redundant transcripts were aligned to public databases. The functions of differential genes were identified by Gene Ontology (GO) enrichment analysis and Kyoto Encyclopedia of Genes and Genomes (KEGG) signal pathway enrichment analysis (significance threshold: E ≤ 10^−5^). StringTie was used to analyze the mRNA expression level by calculating Fragments Per Kilobase of exon model per Million (FPKM) mapped reads (Pertea et al., 2015). The differentially expressed genes (DEGs) were filtered with *p* < 0.05 and | log_2_ (fold change) | > 1.

### qRT-PCR

Quick RNA Isolation Kit (Huayueyang, Beijing, China) was used to extract the total RNA, followed by reverse transcription PCR using the PrimeScript^™^ RT Reagent Kit with gDNA (Takara, Dalian, China). The qRT-PCR was performed in an ABI Step-One Plus Real-Time PCR System (Applied Biosystems, Foster City, CA, United States) using the 2 × SYBR Green qPCR Master Mix (US Everbright Inc., Suzhou, China). The relative expression of each gene was calculated using the 2^–ΔΔCT^ method (Livak and Schmittgen, 2001) using *Csβ-actin* (GenBank: HQ420251) and *CsEF-1α* (XM_028223692.1) as internal controls for normalization. The selected genes were analyzed using three technical and three biological replicates. All primers used in this study are listed in Supplementary Table 1.

### Statistical Analysis

The statistical analysis was performed using SPSS Statistics 20 (IBM, Chicago, United States). Significant differences were determined by one-way ANOVA analysis of variance followed by the least significant difference (LSD) test (*p* < 0.05).

## Results

### General Metabolome Changes Under N Deprivation and Resupply in *Camellia sinensis* Roots

**Figure 1:**
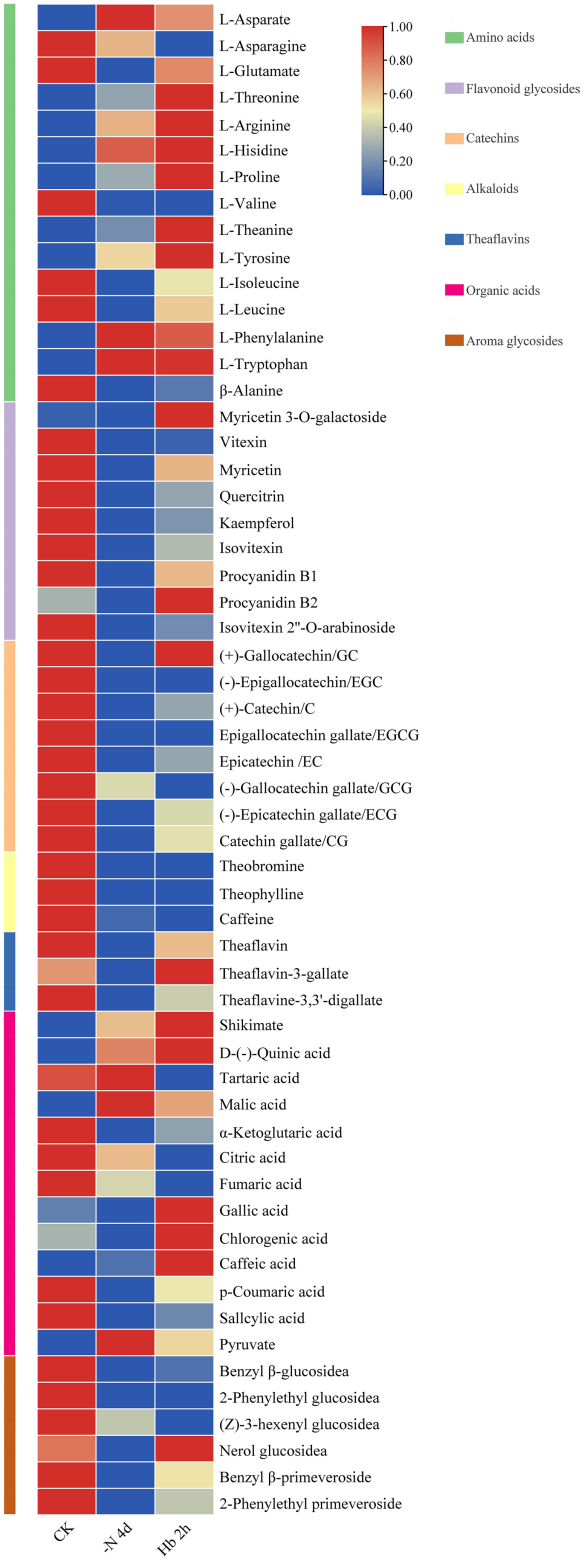
Overall changes of metabolites in *C. sinensis* roots during nitrogen deprivation and resupply. The contents were analyzed with row standardization, with red for higher content and blue for lower content.

The overall metabolomic changes during N deprivation and resupply were explored by non-targeted metabolomic analysis based on the self-built library of metabolic spectrum in our laboratory (Li et al., 2021). A total of 57 compounds, belonging to seven major metabolites, were uncovered (Figure 1; Supplementary Table 2). Specifically, under N deprivation, an overall increase was detected in the content of nine AAs (Figure 1, green group), including aspartate (Asp), threonine (Thr), arginine (Arg), histidine (His), proline (Pro), theanine (Thea), tyrosine (Tyr), phenylalanine (Phen), and tryptophan (Trp). However, the contents of the other six AAs including asparagine (Asn), glutamate (Glu), valine (Val), isoleucine (Iso), leucine (Leu), and alanine (Ala) decreased, but generally rebounded after N resupply. The content of Glu and Ala showed a similar response to N deprivation and resupply, and Thea and Arg exhibited the most abundant relative content. Among the nine flavonoids and flavonoid glycosides detected (Figure 1, purple group), except for myricetin 3-O-galactoside and procyanidin B2, the remaining seven metabolites showed a downtrend, but their content rebounded after N recharge. The contents of eight catechins (Figure 1, orange pink group) and three alkaloids (Figure 1, yellow group) generally descended, with a relative low level in *C. sinensis* roots. Meanwhile, the contents of three theaflavins (Figure 1, blue group) showed a trend of decreasing first and then increasing. Additionally, the contents of over half of the 13 organic acids (Figure 1, plum group) showed an increased tendence, with a relatively high increase in the content of shikimate, the central substance of the shikimate pathway and an important synthetic precursor of aromatic AAs, while a relatively high decrease in the content of α-ketoglutarate, the C skeleton source of Glu family. In terms of aroma glycosides (Figure 1, brown group), the contents of all the six metabolites presented a downtrend during N deficiency, but rebounded for most of them after N resupply. These results suggested that N deficiency affects the biosynthesis of most metabolites in *C. sinensis* roots, and N resupply could restore their synthesis.

### qRT-PCR Validation of RNA-seq Data

**Figure 2:**
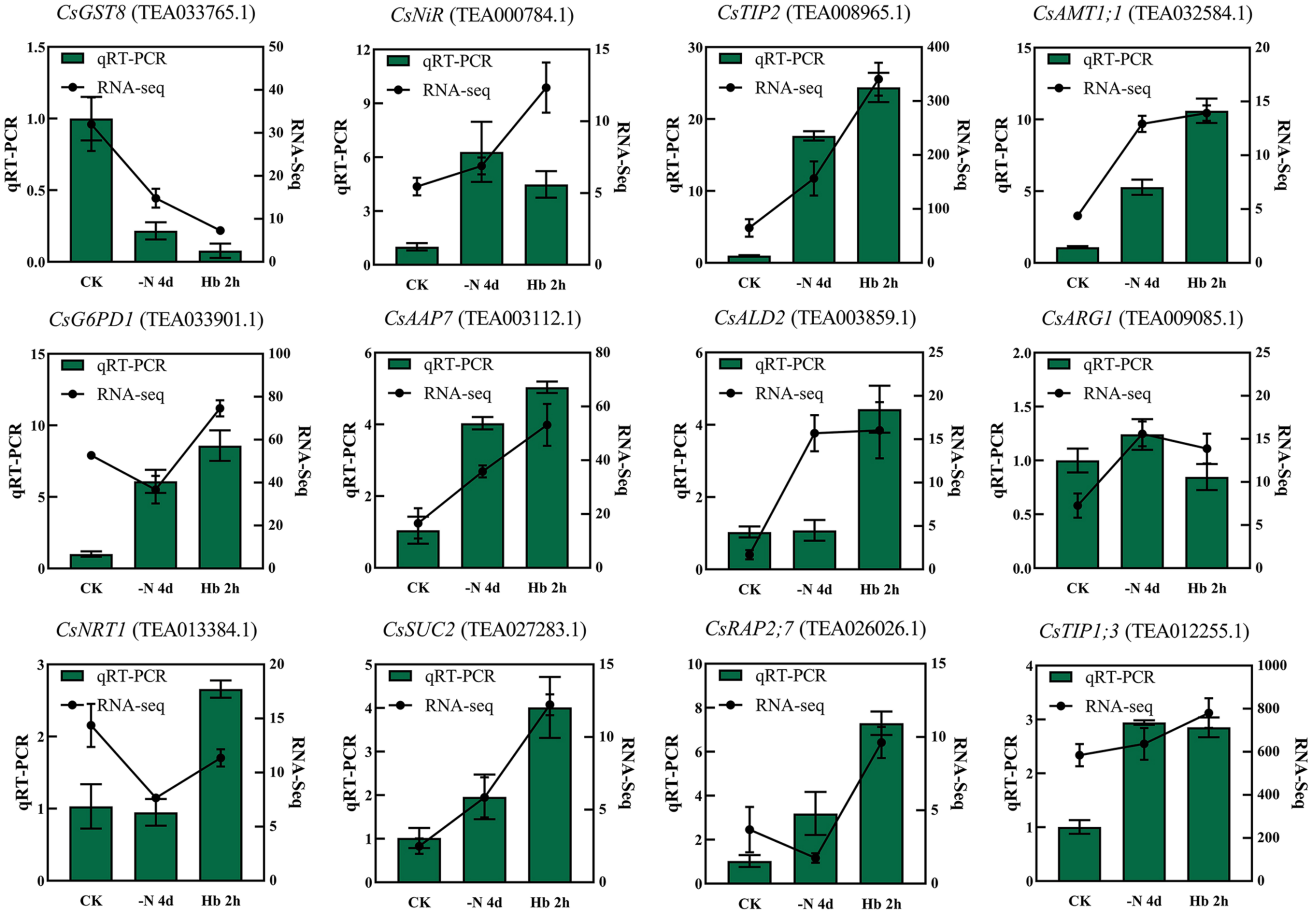
qRT-PCR validation of the expression levels of the 12 randomly selected genes, with the expression level set to 1.0 for each gene in the CK sample.

The accuracy of the RNA-seq results was verified by qRT-PCR analysis of 12 randomly selected genes (Figure 2). Specifically, some of these candidate genes were predicted to be related to glutathione metabolism (*CsGST8* and *CsG6PD1*), N metabolism (*CsNiR*), protein digestion and absorption (*CsAAP7*), lysine biosynthesis (*CsALD2*), and arginine biosynthesis (*CsARG1*). The remaining genes have not been explicitly annotated, but their functions are basically clear, such as aquaporin protein (*CsTIP2* and *CsTIP1;3*), ammonium transporter (*CsAMT1;1*), nitrate transporter (*CsNRT1*), sucrose transport protein (*CsSUC2*), and ethylene-responsive transcription factor (*CsRAP2;7*). The relative expression tendency of the selected genes was generally consistent with the RNA-seq results, indicating the reliability of the RNA-seq data.

### Analysis of Differentially Expressed Genes

For understanding the potential molecular regulatory network, the candidate genes that response to N deprivation and resupply in *C. sinensis* roots were identified by RNA-seq analysis. There were 40 591 reference genes in total. The overall difference of these genes was investigated by differential expression analysis in three libraries in pairs. As a result, 1,480 (with 912 genes up-regulated and 568 down-regulated), 1,549 (with 964 genes up-regulated and 585 down-regulated), and 660 DEGs (with 414 genes up-regulated and 246 down-regulated) were discovered in CK *vs* -N 4d, CK *vs* Hb 2 h, and -N 4d *vs* Hb 2 h, respectively, and 30 DEGs were overlapped among the three groups (Supplementary Figure 1).

### Functional Annotations of DEGs

The biological functions of DEGs were investigated by GO term and KEGG pathway enrichment analysis. In roots, the individual functional terms of DEGs reached 705 (CK *vs* -N 4d *vs* Hb 2 h), 415 (CK *vs* -N 4d), 474 (CK *vs* Hb 2 h), and 306 (-N 4d *vs* Hb 2 h). GO term analysis revealed the enrichment of DEGs mainly in oxidation–reduction process, metal ion binding, oxidoreductase activity, iron ion binding, and extracellular region upon N deprivation and resupply, and metabolic process and transport were also relatively active (Supplementary Figure 2). These results suggested that oxidation–reduction process was very active in the whole N treatment processes. Additionally, many genes were found to be enriched in auxin and cytokinin GO pathways according to transcriptome, and some genes related to abscisic acid, ethylene, and gibberellin were also active.

**Figure 3:**
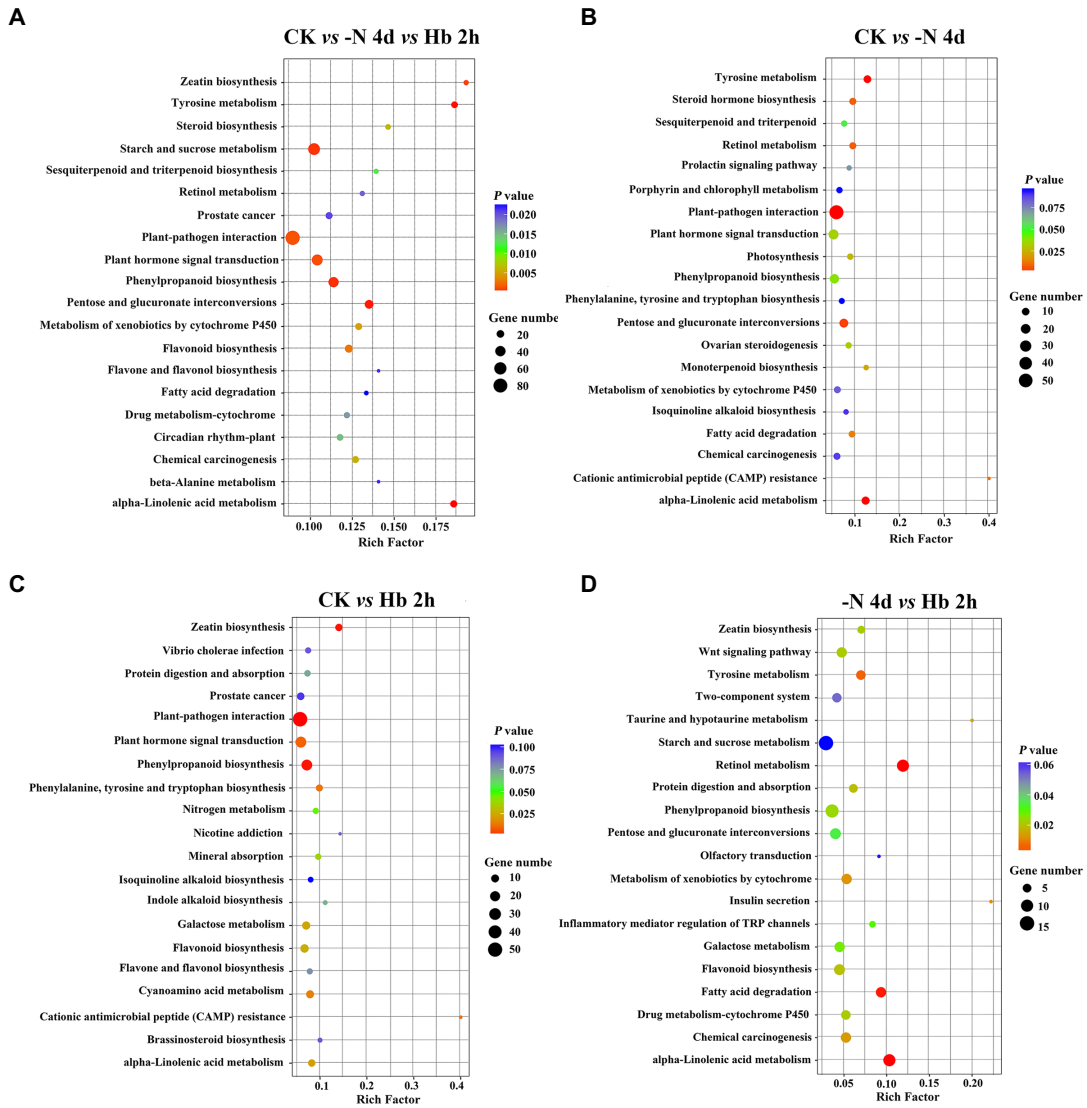
Enrichment of DEGs for the top 20 KEGG pathways in *C. sinensis* roots in CK, -N 4d, and Hb 2 h groups. **(A)** CK *vs* -N 4d *vs* Hb 2 h; **(B)** CK *vs* -N 4d; **(C)** CK *vs* Hb 2 h; **(D)** -N 4d *vs* Hb 2 h. A larger rich factor value denotes a higher degree of enrichment. A lower value of p represents more significant enrichment of the DEGs.

KEGG pathway enrichment analysis can also help to reveal the major metabolic pathways that responses to N deprivation and resupply. Figure 3 showed the top 20 KEGG pathways measured by value of *p* in each group. During N deprivation and resupply, significant changes can be observed in the pathways of tyrosine metabolism, phenylpropanoid biosynthesis, pentose and glucuronate interconversions, alpha-linolenic acid metabolism, and other metabolic pathways. Furthermore, the KEGG pathways related to AAs metabolism and polyphenol metabolism showed a significant response to N stress. In addition, some common important metabolic pathways were also found to response to N stress, such as plant hormone signal transduction, fatty acid degradation, and N metabolism.

Additionally, functional annotation of DEGs revealed that a great portion of transporters, TFs, and genes related to N/AAs metabolism were upregulated under N deprivation and resupply. For transporters, many DEGs classified into ATP-binding cassette (ABC) transporter family, drug/metabolite transporter (DMT) family, and aquaporin (AQP) family. N transporters and AAs transporters have also been found, in which the former mostly falled into peptide transporter family (NPF) and the latter was mostly classified as amino acid permeases (AAPs). For TFs, most of the differentially expressed TFs belonged to WRKY, MYB, NAC, bHLH, and HSF, and for all unigenes screened, a total of 63 kinds TFs were found during N deprivation and resupply, with MYB (140 genes), AP2/ERF-ERF (120), bHLH (113), NAC (100), and WRKY (85) as the top five categories with the largest number of genes. For genes related to N/AAs metabolism, most of them were upregulated especially following N deprivation. The TFs library data was obtained from TPIA (Xia et al., 2019)^2^ and the details of TFs-associated genes were shown in Supplementary Table 3.

### Transcription-Level Regulation of AAs Metabolism in Response to N Deprivation and Resupply in *Camellia sinensis* Roots

**Figure 4:**
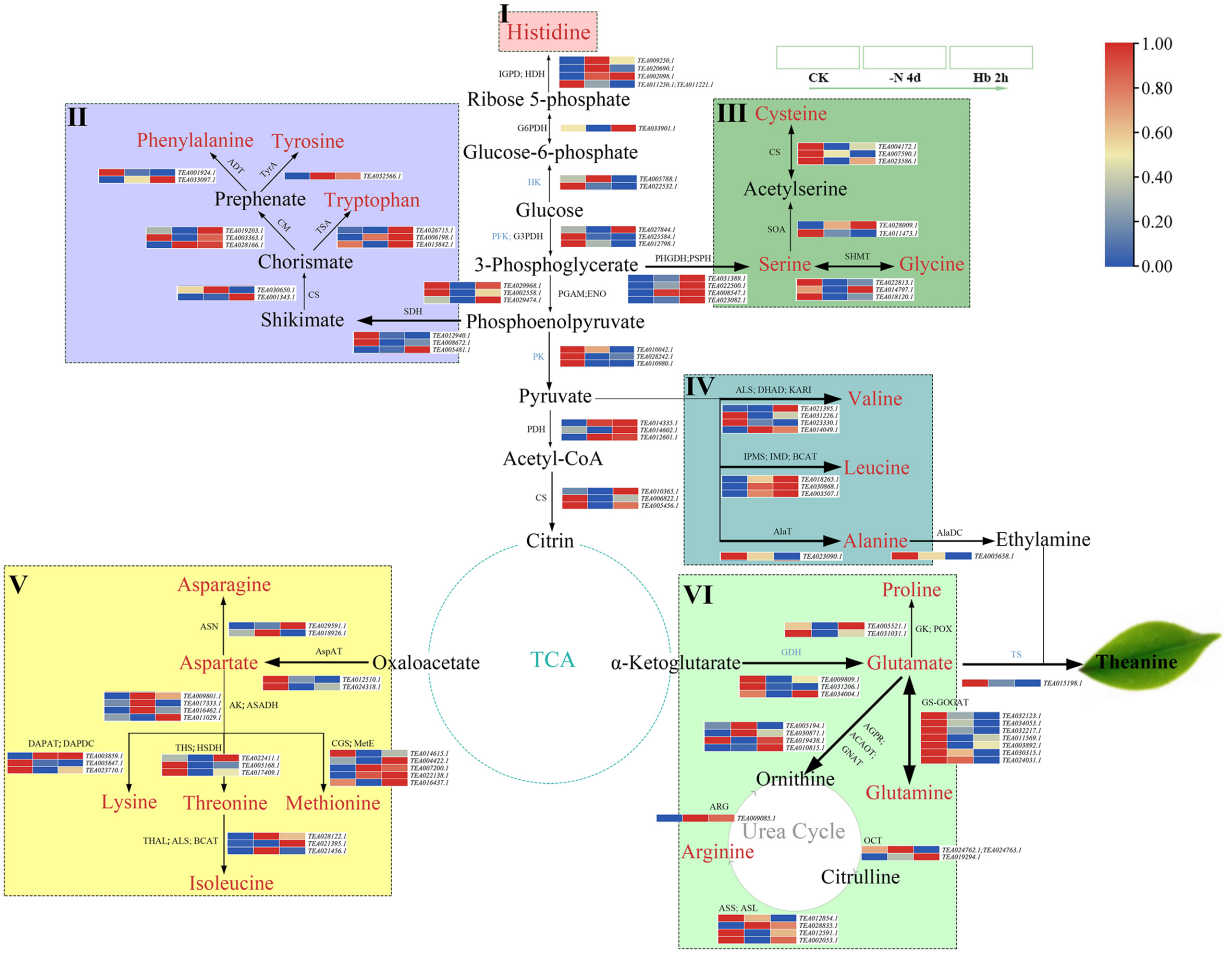
The expression of key genes in the amino acids’ metabolic pathway in *C. sinensis* roots during N deprivation and resupply. I: histidine family (pink frame); II: aromatic AAs family (purple frame); III: serine family (medium-sea-green frame); IV: alanine family (blue frame); V: aspartate family (yellow frame); VI: glutamate family (light-green frame). Relative expression levels are shown by a color gradient from low (blue) to high (red). IGPD, imidazoleglycerol-phosphate dehydratase; HDH, histidinol dehydrogenase; G6PDH, glucose-6-phosphate dehydrogenase; HK, hexokinase; PFK, phosphohexokinase; G3PDH, glyceraldehyde-3-phosphate dehydrogenase; PGAM, phosphoglycerate mutase; ENO, enolase; PK, pyruvate kinase; PDH, pyruvate dehydrogenase; CS, citrate synthase; SDH, shikimate dehydrogenase; CS, chorismate synthase; TSA, tryptophan synthase; CM, chorismate mutase; ADT, arogenate dehydratase; TyrA, arogenate dehydrogenase; PSPH, phosphoserine phosphatase; PHGDH, phosphoglycerate dehydrogenase; SOA, serine O-acetyltransferase; ALS, acetolactate synthase; DHAD, dihydroxy-acid dehydratase; KARI, ketol-acid reductoisomerase; IPMS, 3-isopropylmalate/(R)-2-methylmalate dehydratase small subunit; IMD, isopropylmalate dehydrogenase; BCAT, branched-chain-amino-acid transaminase; AlaT, alanine transaminase; AspAT, aspartate aminotransferase; ASN, asparagine synthase; DAPAT, diaminopimelate aminotransferase; DAPDC, diaminopimelate decarboxylase; THS, threonine synthase; HSDH, homoserine dehydrogenase; THAL, threonine dehydratase; CGS, cystathionine gamma-synthase; MetE, 5-methyltetrahydropteroyltriglutamate-homocysteine methyltransferase; GDH, glutamate dehydrogenase; GK, glutamate 5-kinase; POX, proline oxidase; AGPR, N-acetyl-gamma-glutamyl-phosphate reductase; ACAOT, acetylornithine aminotransferase; GNAT, glutamate N-acetyltransferase; OCT, ornithine carbamoyltransferase; ASS, argininosuccinate synthase; ASL, argininosuccinate lyase; ARG, arginase.

AAs, polyphenols, and caffeine are regarded as three important quality components of tea, and the expression variations of enzyme genes involved in their metabolic pathways were investigated by mapping synthesis-related genes to the KEGG pathway. The AAs metabolism pathway mainly included six AAs families (histidine, aromatic AA, serine, alanine, aspartate, and glutamate, which are presented in the frames with different colors), and they were composed of 20 important AAs, coupled with important metabolic pathways, such as tricarboxylic acid cycle (TCA cycle) and glycolysis cycle (EMP; Figure 4), with the related transcriptome data shown in Supplementary Table 4.

Specifically, His, the only AA of the histidine family (Figure 4 (I), pink frame), with its C skeleton being derived from ribose 5-phosphate in the pentose phosphate pathway (PPP), and the expression of its synthesis-related enzyme genes showed an overall uptrend. In Figure 4 (II) (purple frame), the aromatic AAs family was seen to start from phosphoenolpyruvate (PEP) in the EMP pathway (coupled with erythrose 4-phosphate in the PPP pathway), using shikimate as a synthetic precursor and chorismate as an important bifurcation compound, containing three AAs: Phen, Tyr, and Trp. Almost all the genes involved in the synthesis of the three aromatic AAs showed an uptrend in their expression, which was consistent with the changes of metabolites in *C. sinensis* roots (Figure 1). In Figure 4 (III) (medium-sea-green frame), 3-phosphoglycerate, an intermediate of the EMP pathway was seen to serve as a precursor for the Ser family which consist of Ser and its derivatives Gly and Cys. In this synthesis pathway, the expression of cysteine cynthetase (*CsCS*) and serine hydroxymethyltransferase (*CsSHMT*) showed an overall downtrend, except for serine synthesis. As for the Ala family (Figure 4 (IV), blue frame), it consists of three members: Val, Leu, and Ala, and the expression of Val and Ala generally showed a downtrend, in contrast to an uptrend for Leu. Meanwhile, as an important precursor of theanine and synthesized from Ala, ethylamine was also showed a downregulated expression. For the six AAs of the Asp family (Figure 4 (V), yellow frame), N deprivation induced a downtrend in the expression of the enzyme genes involved in the synthesis of Asp., Lys, and Thr, while Asn, Met, and Iso seemed to be opposite. Interestingly, in the intermediate products of Lys, Thr, and Met synthesis from aspartate, the expression of ASA dehydrogenase (*CsASADH*) and aspartate kinase (*CsAK*) showed a trend of increasing first and then decreasing. For the Glu family (Figure 4 (VI), light-green frame), it contains four AAs, with Glu as an important raw material for Thea synthesis. Additionally, the Glu family can also enter the urea cycle through ornithine. For the synthesis-related enzyme genes in the Glu family, they generally showed a downtrend in expression, especially glutamine-synthetase and glutamate-synthase (GS-GOGAT), which were significantly downregulated after N deprivation. As ethylamine and glutamate were downregulated first, theanine synthetase (*CsTS*; TEA015198.1) showed a lower expression under N deprivation and resupply compared with control. Meanwhile, alanine decarboxylase (*CsAlaDC*), *CsGS*, *CsGOGAT* and *CsTS* showed a high level of expression, probably due to the abundance and response of theanine to N resupply in tea roots, and the expression trend was similar for genes related to the synthesis of Ala, Thea, and ethylamine.

### Transcription-Level Regulation of Polyphenols Metabolism in Response to N Deprivation and Resupply in *Camellia sinensis* Roots

**Figure 5:**
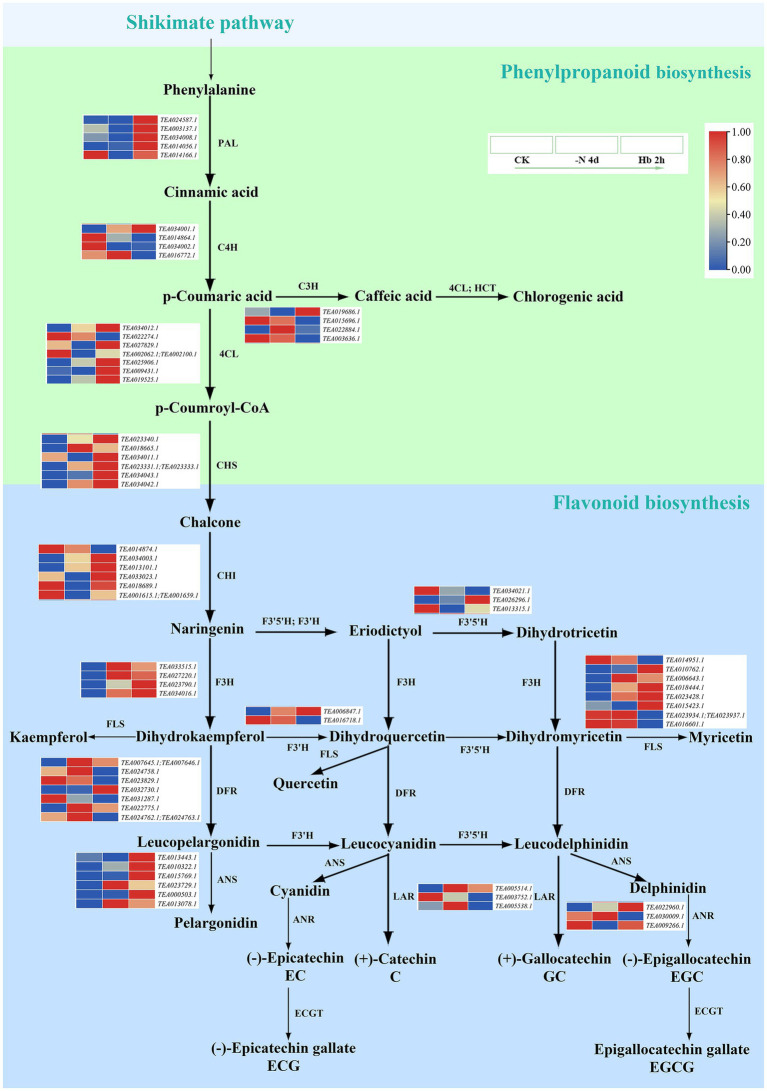
The expression of key genes in the polyphenol metabolic pathway in *C. sinensis* roots during N deprivation and resupply. Relative expression levels are shown by a color gradient from low (blue) to high (red).

In Figure 5, the anabolic pathway of tea polyphenols was seen to initiate from the shikimate pathway, followed by the metabolism of flavonoids (Figure 5, light green) through phenylpropanoid biosynthesis (Figure 5, light blue). The related transcriptome data is shown in Supplementary Table 5. Specifically, naringin synthesized flavonoids and flavonoid glycosides, followed by the synthesis of leucoanthocyanins and anthocyanins, and finally generated catechins. Meanwhile, the fourth polyphenol named organic acid was also formed in the phenylpropanoid biosynthesis pathway through p-coumaric acid. The key genes phenylalanine ammonia-lyase (*PAL*), cinnamate 4-hydroxylase (*C4H*), 4-coumarate-CoA ligase (*4CL*), and chalcone synthase (*CHS*) in the phenylpropanoid biosynthesis pathway can transform phenylalanine into chalcone, followed by catalysis of chalcone isomerase (*CHI*) to form naringin, and the expression of them almost increased except for *C4H*, which was consistent with the KEGG enrichment analysis results (Figure 3). In the conversion reaction of p-coumaric acid to caffeic acid, the expression of cinnamate 3-hydroxylase (*C3H*) was down-regulated. Flavanone 3-dioxygenase (*F3H*), flavanone 3′-dioxygenase (*F3’H*), flavonoid 3′5′-hydroxylase (*F3′5′H*) and flavonol synthase (*FLS*) were mainly involved in the formation of flavonoids and flavonoid glycosides downstream of naringin. The genes that annotated as *F3′H*, *F3′5′H*, and *FLS* were up-regulated or down-regulated in expression, while *F3H* was completely up-regulated. Dihydroflavonol reductase (*DFR*) and anthocyanidin synthase (*ANS*) are responsible for the synthesis of leucoanthocyanins and anthocyanins, respectively, whose expression showed an overall uptrend, and the expression of anthocyanin synthesis-related genes showed a more obvious up-regulation. Leucoanthocyanidin reductase (*LAR*) is involved in the synthesis of catechin (C) and gallocatechin (GC), while anthocyanidin reductase (ANR) catalyzed the formation of epicatechin (EC) and epigallocatechin (EGC). TEA022960.1 that encoded *ANR* was continuously up-regulated during N deprivation and resupply, while the other genes annotated as *LAR* and *ANR* were not. In the present study, we failed to detect genes related to epigallocatechin acyltransferase (*ECGT*) responsible for the synthesis of epicatechin gallate (ECG) and epigallocatechin gallate (EGCG), which may need further research in the future.

### Transcription-Level Regulation of Caffeine Metabolism in Response to N Deprivation and Resupply in *Camellia sinensis* Roots

**Figure 6:**
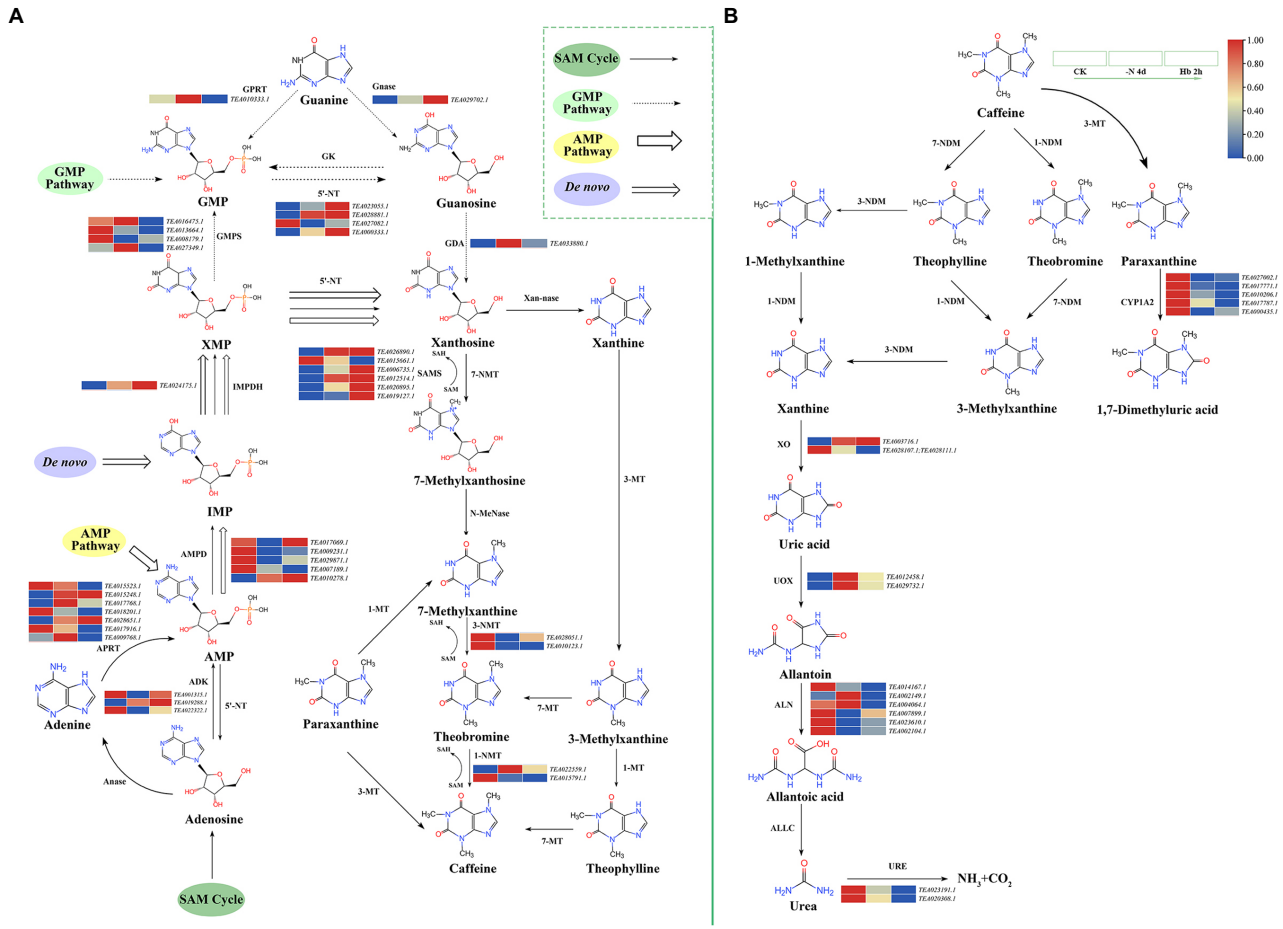
Key gene expressions in the caffeine metabolic pathway in *C. sinensis* roots during *N* deprivation and resupply. **(A)** Caffeine synthesis pathway; **(B)** caffeine degradation. Relative expression levels are shown by a color gradient from low (blue) to high (red). Anase, adenosine nucleosidase; APRT, adenine phosphoribosyltransferase; N-MeNase, N-methyl nucleosidase; Xan-nase, xanthine nucleosidase; MT, methyltransferase; NDM, N-demethylase; ALLC, allantoicase.

Caffeine metabolism pathway includes caffeine synthesis (Figure 6A) and caffeine degradation (Figure 6B), with four pathways for the synthesis of xanthoside in the caffeine synthesis pathway, including S-adenosylmethionine (SAM) cycle (Figure 6, single arrow route), guanine monophosphate (GMP) pathway (Figure 6, dashed arrow route), adenosine monophosphate (AMP) pathway (Figure 6, hollow arrow route), and *De novo* pathway (Figure 6, hollow missing tail arrow route; Ashihara et al., 2017). In caffeine synthesis (Figure 6A), the expressions were mainly downregulated for genes noted as AMP deaminase (*AMPD*), GMP synthetase (*GMPS*), *1-NMT* (methylxanthosine synthase), and *3-NMT* under N deprivation, in contrast to the up-regulated of IMP dehydrogenase (*IMPDH*), guanosine nucleosidase (*Gnase*), 5′-nucleotidase (*5-'NT*), and SAM synthase (*SAMS*) in expression. Meanwhile, no consistent trend was observed in the expression of several genes throughout the experiment, such as the trend of increasing first and then decreasing for guanine phosphoribosyltransferase (*GPRT*) and guanine deaminase (*GDA*), while the trend of decreasing first and then increasing for adenosine kinase (*ADK*). However, the SAM cycle mainly catalyzed by *SMAS* was more active under N stress, providing the methyl donor in the three-step methylation reaction in the synthesis of caffeine. According to research (Zhu et al., 2019), the caffeine and xanthine catabolic pathways were shown to be the two main steps in caffeine degradation (Figure 6B). Specifically, caffeine can be degraded into theophylline, theobromine, and paraxanthine, followed by demethylation reaction to generate xanthine, and finally being decomposed into NH_3_ and CO_2_. The genes encoding cytochrome P450 family 1 subfamily A polypeptide 2 (*CYP1A2*) were suppressed after N deprivation. The two genes (TEA003716.1 and TEA028107.1; TEA028111.1) encoding xanthine oxidase (*XO*) showed an opposite expression trend, while the two genes (TEA012458.1 and TEA029732.1) encoding urate oxidase (*UOX*) displayed a highly similar trend of increasing first and then decreasing. Meanwhile, the genes annotated as allantoinase (*ALN*) and urease (*URE*) showed an overall downtrend in expression. The related transcriptome data is shown in Supplementary Table 6.

### Co-expression Analysis of Genes Related to N, AAs, Polyphenols, and Caffeine Metabolism

**Figure 7:**
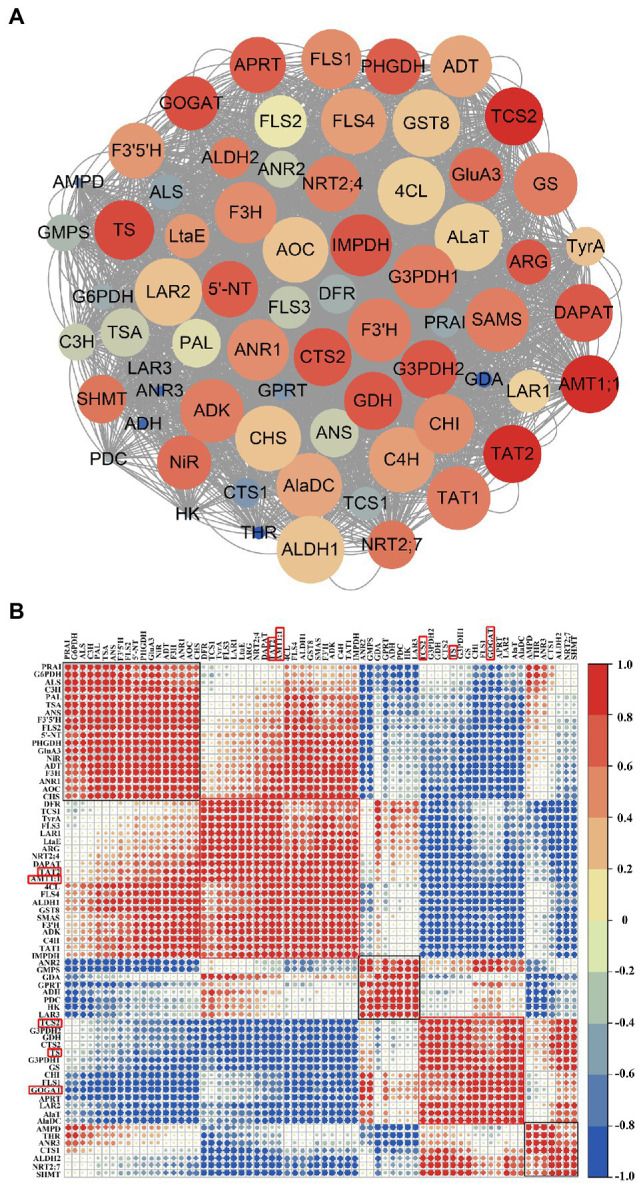
Co-expression network and correlation analysis of genes related to nitrogen metabolism, amino acid metabolism, polyphenol metabolism, and caffeine synthesis. **(A)** Co-expression network visualization by Cytoscape (version 3.9.1), where genes with a high correlation coefficient are marked in “red,” and the circle size indicates the number of genes associated with central gene; **(B)** correlation analysis. Correlation factor (−1 ~ 1) indicates the correlation of two genes at the transcriptional level, with “blue” for negative correlation and “red” for positive correlation, and the larger the circle, the higher the correlation factor.

The related DEGs during N deprivation and resupply were further investigated by co-expression network and correlation analysis of several N-related genes and key enzyme-genes in AAs, polyphenols, and caffeine pathway (Figure 7A). Corrplot package in R (version 4.1.2) was used to determine the correlation coefficient between two genes, and the correlation analysis results of all the selected 68 genes are shown in Figure 7B. In Figure 7A, the co-expression network was seen to consist of 68 nodes and 2,980 edges with coefficient > 0.6 or coefficient < −0.6 between two genes. *AMT1;1*, aminotransferase TAT2 (*TAT2*), *GOGAT*, *TS*, and caffeine synthase (*TCS2*) were identified as key genes, with their correlation coefficient score were the top five among the network. The score was calculated with cytoHubba (Chin et al., 2014) by integrating 11 algorithms. In Figure 7B, the 68 genes were shown to be roughly classified into five categories (enclosed with five square frames) by hierarchical clustering (hclust), with *AMT1;1* and *TAT2* in category 2 and *GOGAT*, *TS*, and *TCS2* in category 4 (red box in Figure 7B), and the two categories showed a negative correlation. In category 1 and 2, most of the genes were related to polyphenol metabolism and N/AAs metabolism; in category 3, three genes were involved in caffeine synthesis (*GMPS*, *GDA*, and *GPRT*) and two genes were related to catechin synthesis (*ANR2* and *LAR3*); in category 4 and 5, the genes were mainly related to AAs and caffeine synthesis. The related data is shown in Supplementary Table 7.

## Discussion

As an essential nutrient, N affects the C and N metabolism of plants, which in turn influences the synthesis of metabolites, as well as plant growth and development. In this study, metabolomics analysis was used to explore the effects of N deficiency and resupply on the main metabolites in tea roots in combination with RNA-seq, and we identified many genes related to N metabolism, AAs metabolism, polyphenols metabolism, caffeine synthesis, and TFs during the treatment, which were responsible for the adaptability of tea roots under N stress (Figure 7; Supplementary Table 7). The effects of N deficit and short-term resupply on plant physiological activities can be observed in variations of metabolites contents, as well as the transcription levels of associated synthase genes (Figures 1, 4–6).

Several studies have shown that the accumulation of metabolites such as AAs in tea plant can be changed under different N conditions, but the response to N deprivation and resupply varies in treatment time and tissues. In this study, roots were selected for experiment because tea plant receives nutrients mainly through its roots, and theanine, an essential and distinct component of tea, is synthesized in tea roots. Meanwhile, the acidity of the root zone was shown to be a major determinant of the N type a plant prefers. Unlike most other plants, tea and rice require acidic pH for development, resulting in their preference for ${\mathrm{NH}}_{4}^{+}$ (Ruan et al., 2007). Additionally, the absorption rate of ${\mathrm{NH}}_{4}^{+}$ by tea plant was reported to higher than that of ${\mathrm{NO}}_{3}^{-}$ in N resupply (Ruan et al., 2021). Gene co-expression analysis also showed that compared with *NRT*, *AMT1;1* was more closely related to *TS* (Figure 7B). In the early stage of N deficiency, plants may take specific strategies for rapid adjustment to N redistribution to ensure normal growth (Huang et al., 2018). Specifically, N deficiency caused pyruvate metabolism to be reprogrammed in soybean (Fataftah et al., 2018). The timing of N resupply can influence the N response and recovery capability of tea plant, as reported previously, AMTs and NRTs in tea plant played leading roles in restoration from N deficiency at 1 and 24 h post N resupply, respectively (Ruan et al., 2021), and pear root can also recover from nitrate deficiency at 24 h post nitrate resupply (Chen et al., 2018).

N is assimilated into various AAs in *C. sinensis* roots, with theanine as the main form of N storage, assimilation, and transport. Histidine family and aromatic AAs family tended to increase in content with upregulation in the expression of genes in their synthetic pathway, largely due to the enhanced activity of their synthetic precursors. Under N deficiency, the expression of *CsAspAT* was downregulated, but the content of aspartate increased, probably because various physiological processes like autophagy (Liu et al., 2021b). Glu family is the main pathway involved in AAs metabolism, providing the substrate Glu for Thea synthesis and exhibiting the strongest response to N deprivation (Yang et al., 2020). The content of Glu initially decreased and then increased during N deprivation and resupply, which is consistent with the expression level of *CsGDH* (Figures 1, 4). Nicotinamide adenine dinucleotide (NADH) can be produced during the synthesis of glutamate from α-ketoglutarate under the catalysis of *CsGDH*, followed by oxidative phosphoric acid reaction to produce a large amount of energy, which may explain the quick recovery of glutamate content after N resupply (Dubois et al., 2003). Glu can be further transformed into glutamine (Gln) and Thea *via* GS-GOGAT and TS, respectively, and N deficit has a stronger influence on the conversion of Glu to Gln, leading to its main conversion to Thea (She et al., 2022), which is consistent with the down-regulation of GS-GOGAT transcription level (Figure 4). *CsTS* was downregulation in transcriptional level but theanine increased slightly in content may attributed to many factors. Firstly, it could be a response to the plant’s own physiological mechanism, it tends to make the energy-producing path more active when in the case of adverse external environment. Besides, the ammonium stored in plant vacuoles can keep its level stable for a short time (Menz et al., 2016). Meanwhile, the 4-day N deprivation may not endanger the survival of tea plant, but allowing low N to promote root growth and enhance theanine synthesis to some extent, and N resupply could even accelerate the synthesis. Secondly, AAs transporters might play a function (Dong et al., 2020), which are heavily involved in theanine transport and storage. Simultaneously, N-related transporters like AMT, are active and reduce ${\mathrm{NH}}_{4}^{+}$ efflux, and TFs like MYB (Zhang et al., 2021) may also take effects. Collectively, the slight increase of theanine content may be attributed to the cooperation of the entire regulatory network in response to N deprivation in *C. sinensis* roots (Kong et al., 2021).

Tea polyphenols and catechins are important components of C pool in tea plant. Under N deficiency and recharge, the content of polyphenols, primarily including flavonoids and catechins, decreased first and then rebounded, indicating that N deficiency had an obvious effect on their synthesis, but they could recover to some extent after N resupply (Figure 1). Most related genes were up-regulated under N deficiency in both phenylpropanoid biosynthesis and flavonoid biosynthesis pathways, suggesting that N deficiency can stimulate their gene expression and promote polyphenol synthesis (Figure 5). Many genes were found to be enriched in flavonoid-related pathway in KEGG analysis (Figure 3). Furthermore, significant enrichment was detected in the GO term of monooxygenase activity in CK *vs* -N 4d (Supplementary Figure 2B), implying that F3’H in polyphenol metabolism may be very active (Jiang et al., 2021). The above data indicated that polyphenol metabolism was very active after N deficiency in *C. sinensis* roots, agreeing with previous results (Huang et al., 2018).

Furthermore, the contents of caffeine, theobromine, and theophylline decreased during N deprivation and resupply, indicating that their syntheses were suppressed by N deprivation, and they had poor adaptability to N resupply. Caffeine synthesis and degradation related-genes showed an overall decrease in expression, implying that caffeine metabolism was not active in N deficiency and resupply, which was consistent with a previous study (Su et al., 2020). This may also be related to the low synthesis of caffeine in *C. sinensis* roots. The caffeine synthesis-related gene *TCS2* was identified as a key gene in N metabolism and was classified into the same category as *TS* and *GOGAT* in co-expression analysis (Figure 7B), which were negatively correlated with the key genes related to N absorption and transport, such as *AMT*, *NRT*, and *NiR* (Ruan et al., 2021), further demonstrating the negative effect of N deficiency on caffeine metabolism.

Various transporters, TFs, and genes related to N/AAs metabolism and hormone were involved in the response to N stress. Transporters and genes involved in N/AAs metabolism help to promote N/AAs metabolism and maintain the normal transport and N cycle (Kong et al., 2021). TFs are also essential for secondary metabolism as well as stress tolerance. MYB can regulate the synthesis of theanine, anthocyanin, and catechin (Zhang et al., 2021; Mei et al., 2021) as well as playing a critical role in response to N stress (Lea et al., 2007), WRKY and bHLH, just like MYB, can also regulate the synthesis and transformation of anthocyanin (Lea et al., 2007; Mei et al., 2021), and NAC can regulate caffeine synthesis (Ma et al., 2022). Furthermore, various hormones synthesized by AAs play important roles in N deficiency stress. Specifically, auxin could accumulate in large quantities to regulate root growth (Hu et al., 2020), and many genes were also enriched in the plant hormone signal transduction KEGG pathway (Figure 4).

## Conclusion

**Figure 8:**
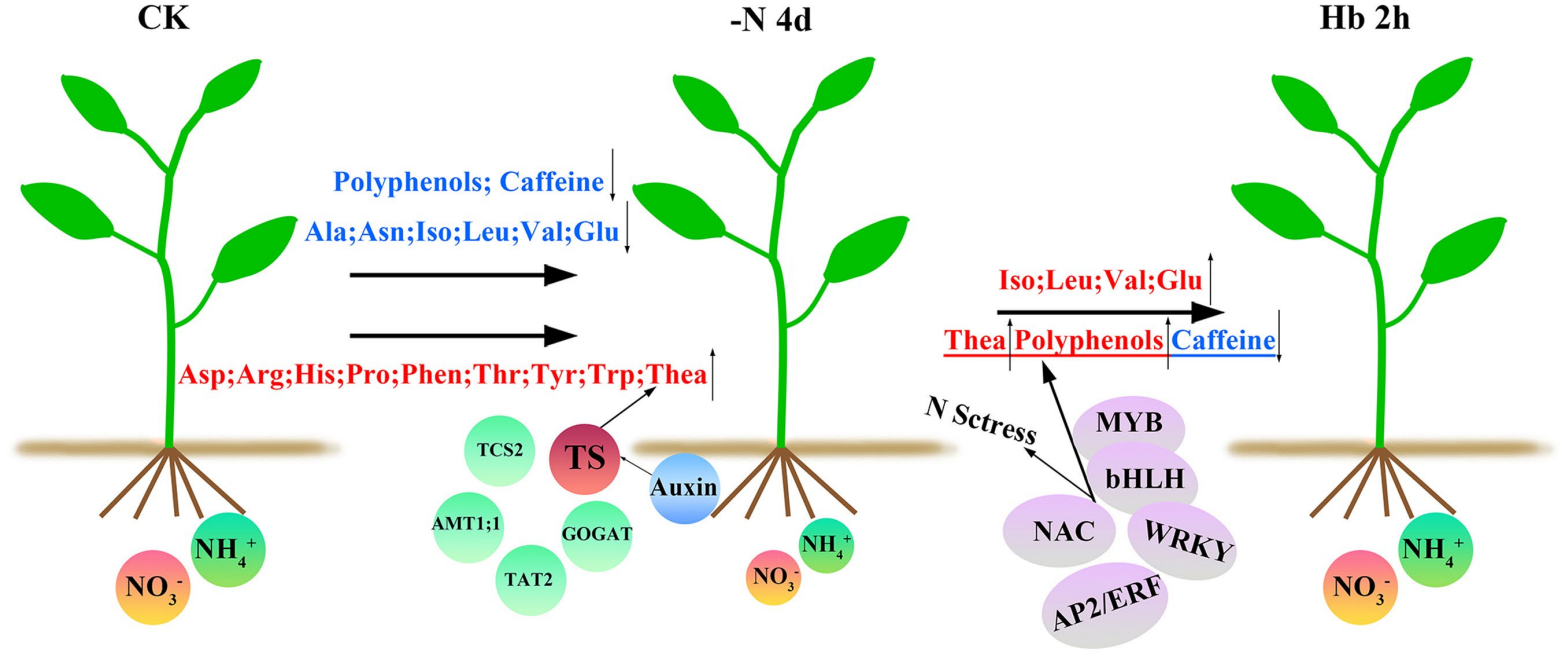
A working model for N-regulation of AAs, polyphenols, and caffeine metabolism during N deprivation and resupply in *C. sinensis* roots. N deficiency resulted in a decrease in some AAs, caffeine, and most polyphenols, but an increase in the other AAs, such as theanine. These results may be influenced by a variety of factors, including the regulation of tea physiological properties, such as a preference for ${\mathrm{NH}}_{4}^{+}$, and the roles of some important transporters, TFs, and hormones in secondary metabolism and stress tolerance. After N recovery, the contents of most down-regulated AAs and polyphenols recovered, but caffeine did not. The substances were shown above and below the arrows, with blue for downregulation and red for up-regulation. *TS* and the four circles around it represent the five key genes screened in this experiment. TFs in the purple ellipse were the most active TFs in N deficiency, which participated in N starvation response and regulated the synthesis of theanine, polyphenols, and caffeine.

In this study, we investigated the global transcriptome change and the expression levels of genes related to AAs, polyphenols, and caffeine biosynthesis pathways of *C. sinensis* roots in response to N deprivation and resupply (Figure 8). N deficiency induced the anabolism of the most AAs, but inhibited the synthesis of the other AAs, polyphenols, and caffeine, and the synthesis of AAs and polyphenols can recover quickly after N resupply, except for caffeine. Additionally, under a short N-deficiency stress, theanine synthesis was maintained which might be due to the cooperation of the entire regulatory network including the physiological regulation mechanism of the tea plant itself, as well as the synergistic action of various TFs, transporters, and hormones. This study helps to shed some light on the molecular mechanism of N regulation in tea plant roots and provides useful genetic information for improving NUE of tea plant.

## Data Availability Statement

The original contributions presented in the study are publicly available. This data can be found at: National Center for Biotechnology Information (NCBI) BioProject database under accession number PRJNA833282.

## Author Contributions

MW conceived and designed the experiment. WX, JL, and LZ performed the experiments. WX wrote the draft. WX, JL, LZ, and XZ analyzed the data. HZ, FG, YW, PW, YC, DN, and MW modified the language of the paper. All authors have read and approved the final version of the manuscript.

## Funding

This work was funded by the National Key Research and Development Program of China (2018YFD1000601 and 2019YFD1001600).

## Conflict of Interest

The authors declare that the research was conducted in the absence of any commercial or financial relationships that could be construed as a potential conflict of interest.

## Publisher’s Note

All claims expressed in this article are solely those of the authors and do not necessarily represent those of their affiliated organizations, or those of the publisher, the editors and the reviewers. Any product that may be evaluated in this article, or claim that may be made by its manufacturer, is not guaranteed or endorsed by the publisher.

## Footnotes

^1^ http://faostat.fao.org

^2^ http://tpdb.shengxin.ren/index.html

## Supplementary Material

Supplementary Table 1 Primers used in this study.

Supplementary Table 2 The relative total amount data of differential metabolites in *C. sinensis* roots during nitrogen deprivation and resupply.

Supplementary Table 3 Statistics of TFs found in *C. sinensis* roots during nitrogen deprivation and resupply.

Supplementary Table 4 The related transcriptome data of key genes expressions in metabolic pathway of amino acids in *C. sinensis* roots during nitrogen deprivation and resupply.

Supplementary Table 5 The related transcriptome data of key genes expressions in metabolic pathway of polyphenols in *C. sinensis* roots during nitrogen deprivation and resupply.

Supplementary Table 6 The related transcriptome data of key genes expressions in metabolic pathway of caffeine in *C. sinensis* roots during nitrogen deprivation and resupply.

Supplementary Table 7 The related data of Co-expression network and correlation analysis.
