# Supplementary material for: Metabolome and RNA-seq Analysis of Responses to Nitrogen Deprivation and Resupply in Tea Plant (Camellia sinensis) Roots
Source: Front Plant Sci. 2022 Aug 26;13:932720. doi: 10.3389/fpls.2022.932720 (PMC9459018; doi:10.3389/fpls.2022.932720)
Supplement: Supplementary Table 1 — Primers used in this study. [file Data_Sheet_1.zip › Supplementary figures.pdf]

Supplementary Figures

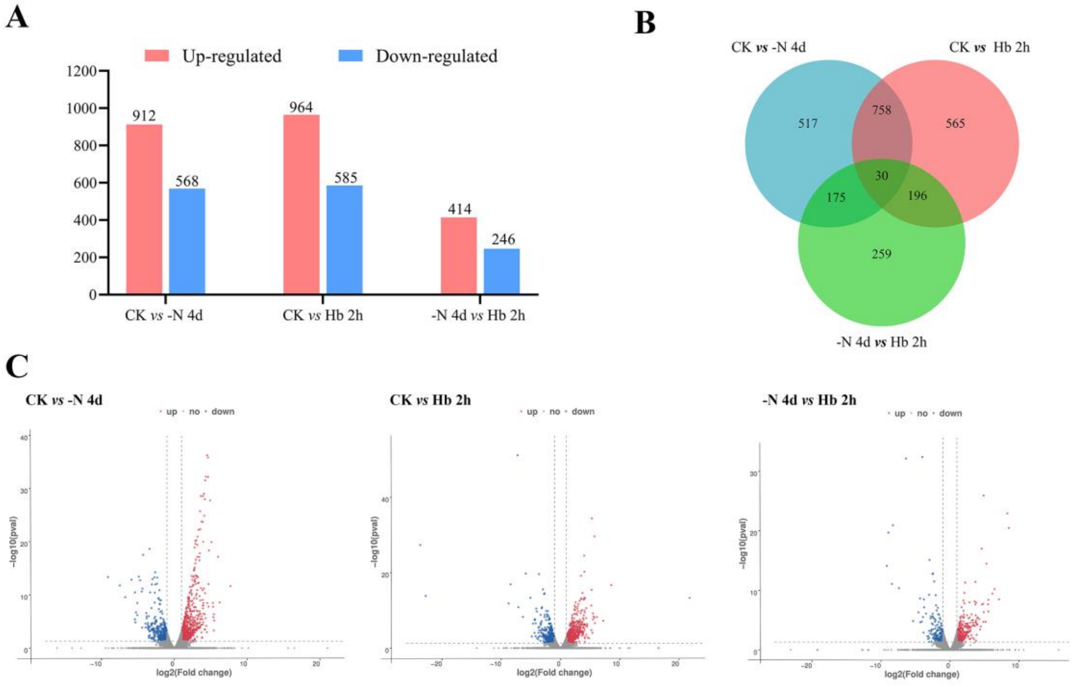

Supplementary Figure 1. DEGs statistics between different groups (CK *vs* -N 4d, CK *vs* Hb 2h, and -N 4d *vs* Hb 2h). (A) The numbers of DEGs between different groups; (B) Venn diagram; (C) Volcano plot.

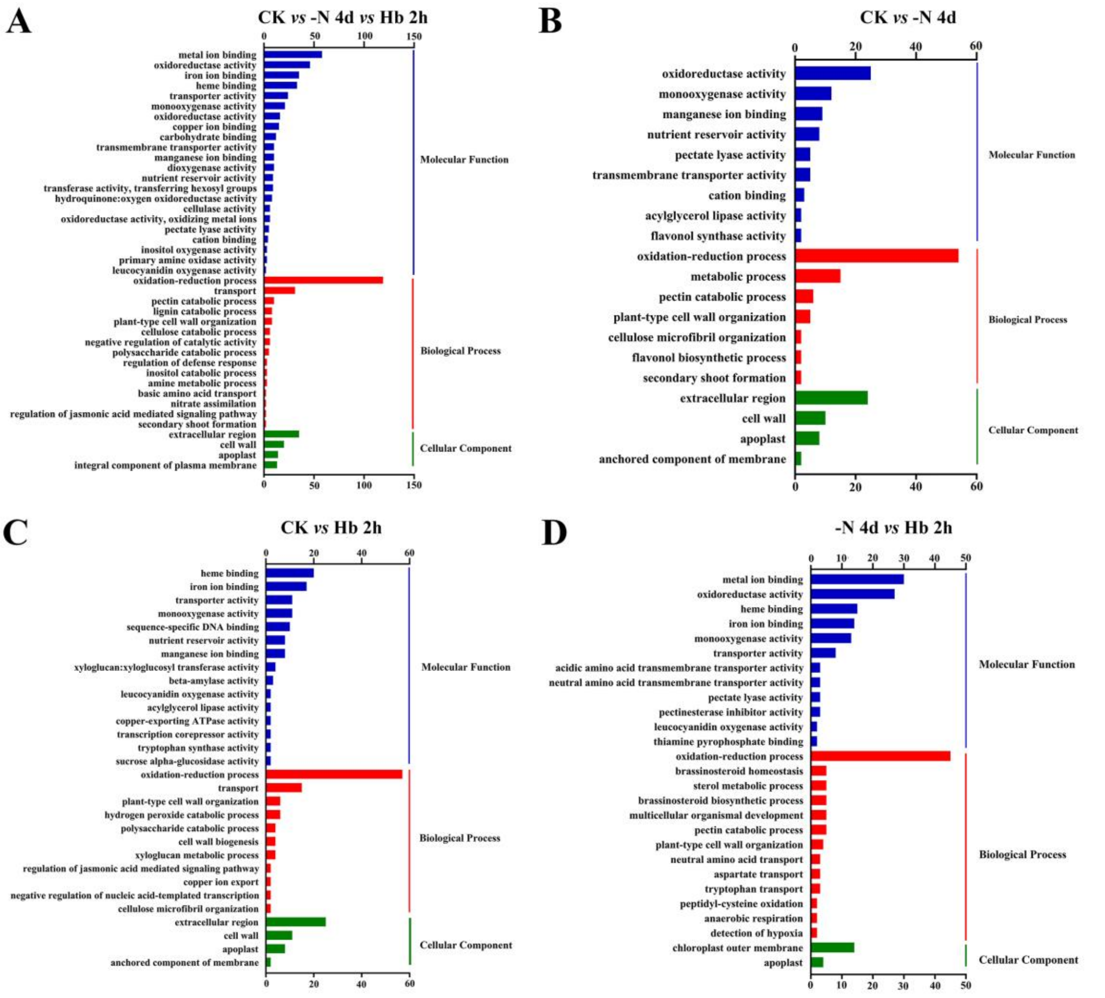

Supplementary Figure 2. GO term analysis of the DEGs between different groups. (A) CK *vs* -N 4d *vs* Hb 2h; (B) CK *vs* -N 4d; (C) CK *vs* Hb 2h; (D) -N 4d *vs* Hb 2h.
